# The time course of health-related Quality of Life in rectal cancer patients undergoing combined modality treatment

**DOI:** 10.1016/j.ctro.2024.100824

**Published:** 2024-07-26

**Authors:** Valentina Tesio, Agata Benfante, Pierfrancesco Franco, Annunziata Romeo, Francesca Arcadipane, Giuseppe Carlo Iorio, Sara Bartoncini, Lorys Castelli

**Affiliations:** a Department of Psychology, University of Turin, Via Verdi 10, 10124, Turin, Italy; b Department of Translational Medicine (DIMET), University of Eastern Piedmont, Via Solaroli 17, 28100, Novara, Italy; c Department of Radiation Oncology, "Maggiore della Carità" University Hospital, Corso Mazzini 18, 28100 Novara, Italy; d Department of Oncology - S. S. Radioterapia, Via Cavour 31, 10123 Turin, Italy; e Department of Oncology - Radiation Oncology, University of Turin, Via Genova 3, 10126 Turin, Italy

**Keywords:** Affectivity, Colorectal cancer, Coping, Longitudinal study, Preoperative treatment, Psychological distress, Quality of life, Rectal cancer, Surgery

## Abstract

**Background and purpose:**

This exploratory prospective observational study investigated the changes in Health-related Quality of Life (HRQoL) in rectal cancer patients (RCPs), from diagnosis to one-year-post-surgery follow-up and explored the role of physical symptoms and psychological determinants on HRQoL at the different time points.

**Materials and methods:**

We assessed HRQoL, psychological distress, coping, affectivity, alexithymia and social support in 43 RCPs treated with preoperative (chemo)radiation and surgery, at three different assessment time points: diagnosis (T0), one month after the end of preoperative treatment (T1), one month after resection surgery (T2), and at follow-up (T3).

**Results:**

The data showed that HRQoL decreased during active treatments, especially between T1 and T2 (*p* = 0.005), before increasing again at follow-up (*p* = 0.002).

Baseline intestinal symptoms (*p* < 0.001) and negative affectivity trait (*p* = 0.03) significantly predicted HRQoL at T0. Baseline pain (*p* < 0.001), intestinal (*p* = 0.003) and urinary (*p* = 0.009) symptoms at T1 significantly predicted HRQoL at T1. A fatalistic coping style at T1 (*p* = 0.013), psychological distress (*p* = 0.003), mouth symptoms (*p* = 0.001) at T2 significantly predicted HRQoL at T2. Similarly, a fatalistic coping style at T1 (*p* = 0.006), psychological distress (*p* = 0.004), mouth (*p* = 0.002) and pain symptoms (*p* = 0.002) at T3 significantly predicted HRQoL at T3.

**Conclusion:**

Several physical and psychological factors are involved in the changes occurring after diagnosis in RCPs’ HRQoL. While cancer-related symptoms and treatment-related physical side effects are the main predictors of HRQoL at diagnosis and during active treatments, early psychological reactions have a higher predictive weight in post-treatment HRQoL.

These data emphasise the importance of active screening, early diagnosis, and preventive psychological interventions immediately after diagnosis to improve HRQoL and psychological health outcomes.

## Highlights

• Prospective study on rectal cancer patients undergoing combined modality treatment.
• HRQoL decreased over time during active combined modality treatment period.
• HRQoL increased at one-year-after-surgery follow-up.
• Physical and psychosocial factors differently weight on HRQoL at different phases.
• Importance of psychological screening both after diagnosis and preoperative therapy.

## 1 Introduction

With around 700,000 new diagnoses per year, rectal cancer represents 30 % of colorectal cancers (CRC), which are the second most common type of cancer worldwide in terms of prevalence and cancer-related mortality in both sexes [1]. The stage of disease contributes to determine the specificities of treatments and its potential consequences. To improve oncologic outcomes and tumor regression, one of the standard treatment options for locally advanced rectal cancer includes preoperative (chemo)radiotherapy, surgical resection (with or without ostomy), and adjuvant chemotherapy for patients with high-risk features [2], [3], [4], [5].

It is undeniable that the diagnosis and treatment of rectal cancer can negatively affect patients’ Health-related Quality of Life (HRQoL), a multidimensional construct that encompasses physical, emotional, cognitive, and social aspects and includes various environmental and personal factors [4], [6], [7], [8], [9], [10].

Psychological distress is associated with a poorer HRQoL [10], [11]. Rectal cancer and consequent cancer therapies can lead to changes in body image and self-representation, as well as fear about treatment outcomes and disease recurrence, which can exacerbate psychological distress such as anxiety and depressive symptoms [10], [11], [12], [13].

Psychological aspects that can influence HRQoL in cancer patients include those related to affective experience and emotion recognition [10], [14]. Positive (e.g., pleasant emotional states, being active, alert, and enthusiastic) and negative (e.g., unpleasant involvement, distress, disgust, guilt) affectivity describe the affective experience and the emotional components of subjective well-being. A high level of positive affectivity promotes psychological well-being and psychosocial adjustment in cancer patients as well as a better HRQoL [10], [15]. In terms of emotion recognition, alexithymia, characterized by difficulties in identifying and describing subjective feelings and bodily sensations, as well as externally oriented thinking, has been associated with poorer health and HRQoL outcomes in populations affected by various medical conditions, including cancer [10], [14], [16], [17].

Coping is a process of self-regulation that involves behavioral and cognitive strategies aimed at managing external and/or internal demands that exceed the individual’s resources, such as cancer-related illness [18], [19]. Cancer patients who choose adaptive styles tend to have better physical health, fewer psychological problems and a better HRQoL [10], [11], [19], [20].

Social support is an important external resource for the individual that positively influences the cognitive adjustment process, with low perception of social support being associated with poorer HRQoL in patients [6], [21], [22].

Although the impact of these psychological aspects on cancer patients HRQoL is known, no studies have specifically analyzed their combined role in the rectal cancer patients (RCPs) or included them in the main category of CRC without providing separate data. In particular, two recent prospective studies investigated HRQoL changes in RCPs treated with different combinations of preoperative chemoradiotherapy, but did not consider psychological predictors or effects [23], [24]. Of the only two studies that looked specifically at psychological distress in RCPs, one examined only psychological distress prior to chemoradiotherapy [13], while the other focused specifically on psychological and sexual distress and did not consider other psychological and clinical variables [12].

For these reasons, this exploratory study aims to evaluate the changes in HRQoL of RCPs during the different treatment phases and at a medium-term follow-up, and to investigate which physical and psychosocial factors better predict HRQoL at the different time points, i.e. after the appointment with the radiation oncologist where patients received the indication for treatment (T0 – diagnosis), after preoperative (chemo)radiotherapy (T1), after surgical resection (T2), and at follow-up one year after surgery (T3).

## 2 Materials and methods

### 2.1 Study design and patient characteristics

This prospective observational cohort study was approved by the Institutional Review Board of the Hospital Ethics Committee (protocol number 0017109, procedure number CS2/1118) and conducted in accordance with the Declaration of Helsinki. Participants were recruited from April 2019 to April 2021 at the “Radiation Oncology Department” of the Hospital “Città della Salute e della Scienza” in Turin, Italy, after providing written informed consent. Inclusion criteria were: age >18-years, a recent diagnosis of rectal cancer, indication for preoperative (chemo)radiotherapy and surgical resection, good knowledge of the Italian, and no severe cognitive or psychopathological disorders as reported in the patients’ medical records. Radiation oncologists recruited patients who met the inclusion criteria, and then referred them to the clinical psychologist to complete the study assessment.

Sociodemographic, clinical, psychological and HRQoL variables were initially collected during the appointment with the radiation oncologist when patients received the indication for treatment (T0 − diagnosis). Psychological (except for alexithymia and trait affectivity) and HRQoL variables were collected again at least one month after the end of preoperative treatment (T1, on average 3 months after diagnosis), at least one month after surgical resection (T2, on average 6 months after diagnosis), and at the follow-up of at least one year after surgical resection (T3, on average 18 months after diagnosis).

### 2.2 Measures

The European Organization for Research and Treatment of Cancer Quality of Life Questionnaire (EORTC-QLQ) C30 (QLQ-C30) and its disease-specific supplementary, the EORTC QLQ-CR29 (QLQ-CR29), were used to assess HRQoL and cancer-related symptoms. In particular, the QLQ-CR29 assesses 4 functional subscales (Body Image (BI), Anxiety (Anx), Weight (Wei), Sexual interest (SexInt)) and 18 symptoms scales, which were grouped in: Urinary Symptoms (UrSy), Intestinal Symptoms (InSy), Pain Symptoms (PainSy), Mouth Symptoms (MoSy), Sexual Symptoms (SexSy). The final score ranges from 0 to 100, with high scores indicating better HRQoL and a favorable outcome on the functional scale, but a greater symptom burden on the symptom scales [25], [26].

The Hospital Anxiety and Depression Scale (HADS) is a 14-items self-report instrument assessing psychological distress symptoms. The HADS total score ranges from 0 to 42, with a cut-off score of 15, with high scores indicating a high level of psychological distress [27], [28].

The Positive and Negative Affect Scale (PANAS) is a self-report instrument on which participants rate the extent to which they experience positive (PA) and negative (NA) affects, from 1 (very slightly) to 5 (extremely). It contains two 10-item versions, one as a trait (PANAS_PAtr and PANAS_NAtr) and one as a state (PANAS_PAst and PANAS_NAst) variable [29].

The Toronto Alexithymia Scale (TAS-20) is a self-report instrument comprising 20 items rated on a five-point Likert scale. The TAS total score ranges from 20 to 100 with a cut-off point ≥61 indicating the presence of alexithymia [30].

The 29-item Mini-Mental Adjustment to Cancer Scale (Mini-MAC) assesses cancer-specific coping styles: cognitive avoidance (CA), fighting spirit (FS), fatalism (F), helplessness/hopelessness (HH) and anxious preoccupation (AP). Responses range from “1 – definitely does not apply to me” to “4 – definitely applies to me” [31].

The Multidimensional Scale of Perceived Social Support Scale (MSPSS) assesses perceived support with 12 items rated on a seven-point Likert scale. Scores range from 12 to 84, with high scores indicating a greater perception of support [32].

### 2.3 Statistical analysis

Statistical analyses were performed using the Statistical Package for Social Sciences − 28.0 (IBM SPSS Statistics for Macintosh, Armonk, NY, USA: IBM Corp.). Descriptive statistics summarized collected variables for the different time points. All variables were normally distributed (absolute values for skewness and kurtosis below 3.0 and 8.0 respectively). The Mann–Whitney *U* test and Fisher’s Exact Test were used for baseline comparisons between completers and dropouts. Repeated-measures analyses were used to assess changes in variables over time, applying the Greenhouse-Geisser correction when sphericity was violated. In case of significant main effects, post-hoc analyses with Bonferroni correction were performed for significant main effects, to assess differences between each time point and the previous one.

Explorative hierarchical multiple regression analyses were performed to investigate which variables better predicted HRQoL (QLQ-C30) at the different time points (T0, T1, T2 and at follow-up). Only significantly correlated variables (Pearson bivariate correlations) were stepwise included in the regression models, in the 1) clinical symptoms (QLQ-CR29 subscales), 2) psychological symptoms (TAS-20, PANAS, HADS, Mini-MAC, MSPSS) and the chronological (first T0, then T1, T2 and then T3) order. Collinearity was assessed using the statistical factors of tolerance and Variance Inflaction Factor (VIF).

## 3 Results

### 3.1 Demographic and clinical characteristics

Forty-three RCPs (two-thirds men) with a mean age of approximately 62 years (range 34–84 years) were enrolled in the study at T0 (Table 1).

**Table 1:** Sociodemographic and clinical characteristics at diagnosis.

		Mean (SD)	N (%)
**Age**		61.6 (12.6)	
**Gender**			
	Male		29 (67.4)
	Female		14 (32.6)
**Educational level** (years)		11.35 (4.3)	
	Primary School		5 (11.6)
	Middle School		12 (27.9)
	High School		15 (34.9)
	Graduate		11 (25.6)
**Marital status**			
	Single/Divorced/Widow(er)		11 (25.6)
	Married/Cohabiting		32 (74.4)
**Work status**			
	Employed		24 (55.8)
	Housewife/Houseman		2 (4.7)
	Retired		17 (39.5)
**TNM Stage**			
Tumor extent:	T3		41 (95.3)
	T4		2 (4.7)
Lymph nodes:	N0		3 (7)
	N1		9 (20.9)
	N2		31 (72.1)
Metastasis:	M0		43 (100)

Most patients were diagnosed as T3N2M0 (8th edition of the TNM staging system) and all but one patient received preoperative chemotherapy in addition to radiotherapy. After surgery, 38 patients (86.5 %) had an ostomy (permanent in 11 patients and temporary in 21 patients) and 17 (39.5 %) patients received adjuvant chemotherapy.

Of the 43 patients enrolled, 3 dropped out at T1, 3 at T2, and 6 at the T3 follow-up for medical or personal reasons. However, the between-group comparisons of sociodemographic, clinical and psychological variables at T0 showed no differences between completers and dropouts.

### 3.2 Clinical and psychological changes over time

Table 2 shows T0 (N = 43), T1 (N = 40), T2 (N = 37) and T3 (N = 31) descriptive data and the p values of the repeated measures ANOVAs, assessing the main effect of time for each variable.

**Table 2:** Repeated measures ANOVAs on Health-Related Quality of Life (QLQ-C30) at diagnosis (T0), after preoperative treatments (T1), after surgical resection (T2)) and at follow-up (T3; N = 31).

	T0	T1	T2	T3		
	N = 43	N = 40	N = 37	N = 31	F(df1,df2)	*p*
**QLQ-C30**	86.89 (8.9)	87.20 (12.3)	80.52 (12.8)	87.25 (10.5)	F(2.4,72.4) = 5.87	0.003
**QLQ-CR29**						
QLQ-CR29_BI	92.51 (12.8)	86.11 (14.8)	78.38 (19.4)	78.85 (21.9)	F(2.4,73.1) = 6.93	<0.001
QLQ-CR29_Anx	46.51 (28.3)	65 (25)	68.47 (26)	69.89 (24.9)	F(3,90) = 7.92	<0.001
QLQ-CR29_Wei	89.15 (21.5)	85.83 (19.8)	84.68 (21.7)	86.02 (22.4)	F(3,90) = 0.68	0.566
QLQ-CR29_SexInt	25.58 (28)	30.83 (26.6)	13.51 (22.9)	22.58 (26.4)	F(2.1,63.9) = 7.72	<0.001
QLQ-CR29_SexSy	10.08 (18.6)	19.66 (30.3)	26.13 (36.1)	29.03 (37.3)	F(2.42,70.2) = 3.23	0.037
QLQ-CR29_UrSy	9.82 (13)	13.19 (15)	16.97 (16.6)	8.78 (11.5)	F(2.49,74.69) = 6.42	0.001
QLQ-CR29_InSy	17.21 (13.8)	11.75 (12.7)	14.96 (13.2)	14.62 (13.7)	F(2.23,66.8) = 0.64	0.547
QLQ-CR29_PainSy	19.38 (16.9)	14.58 (14)	20.27 (13.8)	16.4 (12.1)	F(2.37,71.15) = 1.41	0.249
QLQ-CR29_MoSy	9.69 (13.2)	12.08 (16.9)	17.12 (20.2)	9.68 (15.4)	F(3,90) = 3.59	0.017
**TAS-20**	44.81 (10.5)					
**PANAS**						
PANAS_PAtr	36.98 (6.2)					
PANAS_NAtr	18.93 (5.8)					
PANAS_PAst	31.86 (6.4)	31.8 (6.1)	30.89 (5.9)	34.58 (6.7)	F(3,90) = 5.59	0.001
PANAS_NAst	18.38 (6.3)	16.22 (5.8)	15.89 (5.7)	16.32 (6.5)	F(2.2,66) = 2.13	0.123
**HADS**	10.07 (5.3)	7.67 (5.3)	9.81 (6.2)	8.45 (5.9)	F(2.47,74) = 3.14	0.039
**MSPSS**	73.23 (10.7)	73.08 (9.3)	71.46 (12.1)	69.42 (12.4)	F(3,90) = 2.96	0.036
**Mini-MAC**						
Mini-MAC_F	2.93 (0.62)	2.96 (0.6)	2.96 (0.6)	3 (0.47)	F(3,90) = 0.19	0.901
Mini-MAC_FS	3.38 (0.44)	3.39 (0.5)	3.3 (0.4)	3.14 (0.35)	F(3,90) = 3.58	0.017
Mini-MAC_HH	1.56 (0.44)	1.54 (0.5)	1.54 (0.5)	1.51 (0.39)	F(3,90) = 0.09	0.965
Mini-MAC_AP	2.63 (0.61)	2.39 (0.6)	2.15 (0.6)	2.25 (0.53)	F(3,90) = 10.66	<0.001
Mini-MAC_CA	2.78 (0.76)	2.76 (0.7)	2.7 (0.8)	2.77 (0.61)	F(3,90) = 0.35	0.790

QLQ-CR29: EORTC colorectal cancer module:_BI: Body Image,_Anx: Anxiety,_Wei: Weight,_SexInt: Sexual Interest, functional scales;_SexSy: Sexual Symptoms;_UrSy: Urinary Symptoms,_InSy: Intestinal Symptoms,_PainSy: Pain Symptoms,_MoSy: Mouth Symptoms, subcales; TAS-20: Toronto Alexithymia Scale; PANAS: Positive and Negative Affect Scale,_PAtr: Positive Affect Trait;_NAst: Negative Affect Trait;_PAst: Positive Affect State;_NAst: Negative Affect State; HADS: Hospital Anxiety and Depression Scale; MSPSS: Multidimensional Scale of Perceived Social Support; Mini-MAC: Mini-Mental Adjustment to Cancer scales,_F: Fatalism,_FS: Fighting Spirit,_HH: Helplessness/Hopelessness,_AP: Anxious Preoccupation,_CA: Cognitive Avoidance.

The QLQ-C30 showed high-to-medium average scores, suggesting an overall preserved HRQoL, which, however, decreased over time during active treatments (post-hoc contrasts showed a statistically significant decrease between T1 and T2: F(1,30) = 7.65, *p* = 0.010), and then improved again at follow-up (post-hoc contrast T2 vs. T3: F(1,30) = 11.35, *p* = 0.002).

The functional subscales of the QLQ-CR29 revealed a statistically significant change in the QLQ-CR29_BI and QLQ-CR29_SexInt scales: the body image decreased between T0 and T1 (F(1,30) = 8.94, *p* = 0.006), whereas sexual interest decreased between T1 and T2 (F(1,30) = 12.55, *p* = 0.001) and then improved between T2 and T3 (F(1,30) = 6.23, *p* = 0.018).

The QLQ-CR29_Anx showed an improvement over time, with post-hoc contrasts showing a significant decrease in health anxiety between T0 and T1 (F(1,30) = 10.89, *p* = 0.002).

The CLQ-CR29 also revealed an overall low level of symptoms, with QLQ-CR29_UrSy, QLQ-CR29_MoSy and QLQ-CR29_SexSy changing over time. Post-hoc contrasts showed that: urinary symptoms significantly worsen between T0 and T1 (F(1,30) = 6.69, *p* = 0.015) and then improved between T2 and T3 (F(1,30) = 10.39, *p* = 0.003); sexual symptoms worsen between T0 and T1 (F(1,29) = 5.21, *p* = 0.030); mouth area symptoms significantly improved between T2 and T3 (F(1,30) = 9.42, *p* = 0.005).

In terms of psychological traits, we found a low level of alexithymia, with only 6 (14 %) patients scoring above the TAS-20 cut-off, and a low tendency to experience a negative affectivity (PANAS_NAtr). The PANAS_NAst did not change over time, while the PANAS_PAst statistically increased between T2 and the T3 follow-up (post-hoc contrast: F(1,30) = 16.56, *p* < 0.001).

The HADS showed a fluctuating trajectory of psychological distress symptoms, with post-hoc contrasts showing a statistically significant decrease between T0 and T1 (F(1,30) = 11.64, *p* = 0.002), followed by a subsequent increase between T1 and T2 (F(1,30) = 7.55, *p* = 0.010).

The MSPSS indicates a very high level of perceived social support at all the assessment time points. The Mini-MAC showed that Fighting Spirit and Helplessness/Hopelessness were the most and least utilized coping styles, respectively, while Anxious Preoccupation statistically decreased over time (post-hoc contrasts: T0 vs. T1: F(1,30) = 8.19, *p* = 0.008; T1 vs. T2: F(1,30) = 5.17, *p* = 0.030).

### 3.3 Explorative regression analyses

**Fig. 1:**
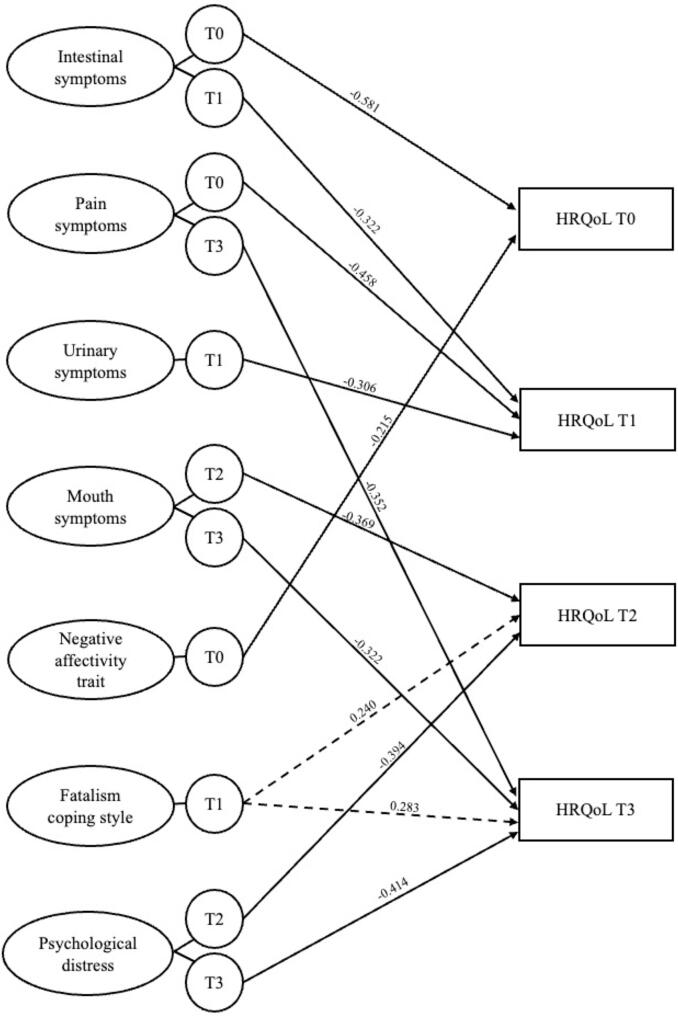
Final models of the explorative hierarchical multiple regressions on the Health-Related Quality of Life (QLQ-C30) at the different time points. Only statistically significant predictors were shown with their β-value. The dashed line indicates the only positive predictive factor.

Correlation analyses between HRQoL at T0, T1, T2 and T3 and all other variables were performed to identify the variables to be included in the explorative hierarchical multiple regression models (Supplemental Appendix 1). The full regression models are presented in Supplemental Appendix 2, 3, 4 and 5, and the final models are summarized in Table 3 and Fig. 1.

**Table 3:** Hierarchical multiple regressions with Health-Related Quality of Life (QLQ-C30) at the different times as dependent variables.

	Predictor	R^2^	Adj R^2^	F	F-ΔR^2^	B	SE B	*β*	*p*
***QLQ-C30 at T0***									
**4**	*(Constant)*	0.71	0.68	23.33***	5.05*	*98.93*	*3.49*		*<0.001*
	QLQ-CR29_InSy_T0					−0.38	0.07	−0.581	<0.001
	QLQ-CR29_PainSy_T0					−0.11	0.06	−0.199	0.082
	QLQ-CR29_Anx_T0					0.06	0.03	0.185	0.054
	PANAS_NAtr_T0					−0.33	0.15	−0.215	0.030

***QLQ-C30 at T1***									
**4**	*(Constant)*	0.82	0.80	40.93***	7.58**	*101.17*	*1.42*		*<0.001*
	QLQ-CR29_PainSy_T0					−0.33	0.06	−0.458	<0.001
	QLQ-CR29_UrSy_T0					−0.06	0.11	−0.063	0.569
	QLQ-CR29_InSy_T1					−0.31	0.10	−0.322	0.003
	QLQ-CR29_UrSy_T1					−0.25	0.09	−0.306	0.009

***QLQ-C30 at T2***									
**5**	*(Constant)*	0.75	0.71	18.97***	10.65**	92.21	8.45		*<0.001*
	TAS-20					−0.18	0.13	−0.143	0.181
	PANAS_NAst_T0					−0.39	0.20	−0.195	0.056
	Mini-MAC_F_T1					5.15	1.95	0.240	0.013
	QLQ-CR29_MoSy_T2					−0.23	0.07	−0.369	0.001
	HADS_T2					−0.81	0.25	−0.394	0.003

***QLQ-C30 at follow-up (T3)***									
**6**	*(Constant)*	0.84	0.80	20.78***	10.16**	81.67	8.66		<0.001
	QLQ-CR29_Anx_T1					−0.02	0.04	−0.053	0.627
	PANAS_PAst_T1					0.19	0.18	0.116	0.291
	Mini-MAC_F_T1					4.82	1.59	0.283	0.006
	QLQ-CR29_PainSy_T3					−0.31	0.09	−0.352	0.002
	QLQ-CR29_MoSy_T3					−0.22	0.06	−0.322	0.002
	HADS_T3					−0.74	0.23	−0.414	0.004

*p-value < 0.05; ** *p*-value <0.01; ***p-value < 0.001.

QLQ-CR29: EORTC colorectal cancer module:_InSy: Intestinal Symptoms,_PainSy: Pain Symptoms,_Anx: Anxiety;_UrSy: Urinary Symptoms;_MoSy: Mounth Symptoms; PANAS: Positive and Negative Affect Scale:_NAtr:_Negative Affect trait scale;_NAst:_Negative Affect state scale;_PAst:_Positive Affect state scale; TAS-20: Toronto Alexithymia Scale; Mini-MAC_F: Mini-Mental Adjustment to Cancer scales_Fatalism; HADS: Hospital Anxiety and Depressive Scale.

Regarding QLQ-C30 at T0, QLQ-CR29_InSy at T0 (*β* = −0.581, *t*(38) = −5.67, *p* < 0.001) and trait negative affect (PANAS_NAtr_T0: *β* = −0.215, *t*(38) = −2.25, *p* = 0.030) were the only statistically significant negative predictive factors, with the final model explaining 68 % of the variance (QLQ-C30_T0: F(4,38) = 23.3, *p* < 0.001).

Regarding QLQ-C30 at T1, QLQ-CR29_PainSy at T0 (*β* = −0.458, *t*(35) = −5.31, *p* < 0.001), QLQ-CR29_InSy at T1 (*β* = −0.322, *t*(35) = −3.25, *p* = 0.003) and QLQ-CR29_UrSy at T1 (*β* = −0.306, *t*(35) = −2.75, *p* = 0.009) were the statistically significant predictive factors, with the final model explaining 80 % of the variance (QLQ-C30_T1: F(4,35) = 40.93, *p* < 0.001).

With respect to QLQ-C30 at T2, the final model explained 71 % of the variance (QLQ-C30_T2: F(5,31) = 18.97, *p* < 0.001). Psychological distress at T2 (HADS_T2: *β* = −0.394, *t*(31) = −3.26, *p* = 0.003) was the strongest negative contributor, followed by QLQ-CR29_MoSy at T2 (*β* = −0.369, *t*(31) = −3.56, *p* = 0.001) and the Mini-MAC_F at T1 (*β* = 0.040, *t*(31) = 2.65, *p* = 0.013). The latter was the only positive predictive factor: the more the patients adopted a fatalistic coping style at T1, the better their HRQoL at T2.

Similarly, QLQ-C30 at the T3 follow-up was significantly predicted by psychological distress (HADS_T3: *β* = −0.414, *t*(30) = −3.19, *p* = 0.004), QLQ-CR29_MoSy (*β* = −0.322, *t*(30) = −3.55, *p* = 0.002) and QLQ-CR29_PainSy (*β* = −0.352, *t*(30) = −3.42, *p* = 0.002) at T3 and by the Mini-MAC_F at T1 (*β* = 0.283, *t*(30) = 3.04, *p* = 0.006).

## 4 Discussion

The aim of the present exploratory longitudinal study was to evaluate changes in HRQoL of RCPs during cancer treatment, i.e. after diagnosis, after preoperative (chemo)radiotherapy and after surgical resection, and at a medium term follow-up (one years after surgery), and to assess which physical and psychosocial factors better predict HRQoL in the different time points. Deepening the understanding of the positive and negative predictive factors for patients’ HRQoL at different phases could improve screening programs for early detection and intervention.

Most previous studies referred to the broader population of CRC patients and did not provide separate data focusing RCPs. Therefore, we performed the T0 assessment on 43 RCPs who had just received the diagnosis and treatment program. Consistent with two recent studies of CRC patients [33], [34], HRQoL was preserved at this time point and patients had few physical symptoms. The main symptoms included intestinal and pain symptoms such as blood and mucus in the stool, flatulence, high stool frequency and buttock pain, which were directly associated with locally advanced rectal cancer [2], [3]. Overall preserved HRQoL at T0 was associated with moderate levels of psychological distress, as in the only other study that examined psychological distress in RCPs before starting active treatments [13]. In our study, RCPs after diagnosis also showed high levels of health anxiety. The high level of health anxiety and psychological distress may be due to the initial burden due to cancer diagnosis and concern about the side effects of preoperative treatments, in particular those related to radiotherapy, which cancer patients are least aware of [35], [36].

The most recent longitudinal studies in CRC patients suggested an improvement in HRQoL over time [33], [34], [37]. However, all of these studies recruited patients who had already undergone major cancer treatments [33], [34], [37]. When assessing changes since diagnosis, our data showed that HRQoL deteriorated significantly during the active treatment phases, particularly after surgery, before improving again at medium-term follow-up. These results are consistent with the only studies we are aware of comparing HRQoL of CRC patients [38] and RCPs [23] before and after surgery, which showed similar deterioration after surgery and subsequent improvement after one year.

The decline in HRQoL during active treatment came with a functional deterioration in body image and a general symptoms’ worsening over the course of treatments, particularly in relation to the urinary system, the mouth area, and sexual symptoms (QLQ-CR29). Specifically, urinary and sexual symptoms increased after preoperative (chemo)radiotherapy as a possible side effect, while sexual interest worsened after surgery, probably due to the consequences of resection [4], [7], [8]. Although physical symptoms increased, health anxiety improved over time, decreasing significantly after preoperative (chemo)radiotherapy. This improvement after preoperative (chemo)radiotherapy could further suggest that the high level of health anxiety at diagnosis could be partially due to the worry about the effects of radiotherapy [35], [36]. Similarly, the use of the Anxious Preoccupation coping style decreased over the course of the active treatments.

Also psychological distress decreased after preoperative treatments, but it increased again after surgery, probably due to adjustment to postoperative conditions (e.g., ostomy management) or to eventual adjuvant therapy [12], [34], [37]. The only other study that assessed psychological distress in RCPs prior to preoperative treatment reported an overall decrease in psychological distress over time, although the mean scores seemed to confirm our fluctuating trend [12].

At follow-up, after the functional deterioration and worsening of symptoms that occurred during the course of treatment, there was a general improvement with a reduction in urinary and mouth area symptoms and a functional improvement in body image and sexual interest. In terms of psychosocial variables, psychological distress did not change significantly between T2 and follow-up, but patients experienced an increase in positive affect. The overcoming of the active treatment phase and the reduction of the side effects of those treatments leads to a progressive improvement in physical and mental health which results in an improvement in the HRQoL [34].

The explorative analyses conducted to evaluate possible positive and negative predictive factors suggested that physical and psychosocial factors have a different weight in impacting HRQoL during the different phases. At diagnosis, intestinal symptoms and trait negative affect negatively predicted HRQoL. After preoperative treatments, HRQoL was significantly explained by intestinal and urinary symptoms at that time point and by the pain symptoms experienced at diagnosis. After surgery, HRQoL was significantly explained by psychological distress and mouth symptoms at that time point, and by the adoption of the fatalism coping style after the preoperative treatment. Similarly, at follow-up, HRQoL was mainly explained by psychological distress and residual clinical symptoms at that time point (in particular, pain and mouth area symptoms), and by the adoption of the fatalism coping style after the preoperative treatment.

On the one hand, these data confirm the strong influence of physical symptoms on HRQoL in RCPs [8], [38]. However, this seems to be particularly the case at diagnosis and during active treatments, when cancer-related symptoms (i.e., intestinal and/or pain symptoms) and treatment-related physical side effects are the most important predictive factors. On the other hand, the data suggest that although psychological variables appear to have a smaller concurrent effect in the early phases, psychological reaction at these early phases has a higher weight in predicting RCPs’ HRQoL after active treatments and at medium-term. Indeed, a greater use of fatalism after preoperative (chemo)radiotherapy positively predicted HRQoL after surgery and at the one-year-after-surgery follow-up. This tendency towards a resigned and stoic attitude towards the disease and an external locus of control prior to surgery could be an indicator of greater acceptance and confidence in treatment, which could then translate into better HRQoL outcomes [10], [11], [19], [20]. In contrast, greater difficulty in acceptance and adaptation, which may also result in the persistence of high levels of psychological distress after surgery, becomes the factor that plays a greater role in explaining HRQoL after the end of active treatments and at medium-term, along with long-term treatment-related side effects (such as pain and mouth symptoms).

### 4.1 Study limitations

The main limitation of the present study is the small sample size, which reduces the power of the analyses, potentially affecting some of the findings of the study. The COVID-19 pandemic not only hindered the recruitment and subsequent reassessment of patients, but also made access to combined-modality cancer treatments more difficult, leading to a decrease in the number of patients. Future longitudinal studies with a larger sample of RCPs are needed to further assess the impact of rectal cancer and the different treatments on patients’ QoL.

### 4.2 Clinical implications

From a clinical perspective, our findings emphasise that multiple physical and psychological factors play a role in the changes in patients’ HRQoL in response to cancer diagnosis and treatments. Overall, these data suggest the need for bio-psycho-social assessment of RCPs from the communication of diagnosis, through all subsequent phases of the treatment process to follow-up, as each phase has physical and psychological specificities. Based on these specificities, support services should be tailored to both the individual patient and the treatment phase, in particular by implementing multidisciplinary and multimodal preventive and pre-habilitation interventions not only before surgery [39], [40], but even better immediately after diagnosis to improve both cancer-related reactions and HRQoL and psychological health in the medium term.

### 4.3 Conclusions

The findings of this study showed an overall worsening of HRQoL in RCPs from diagnosis to one month after surgical resection and an improvement from that time to follow-up, one year after surgery. In addition to surgery, preoperative (chemo)radiotherapy seemed to be a crucial step from both a psychological and physical point of view. This is because not only the side effects, especially those related to the urinary system, are among the physical symptoms that significantly worsen HRQoL one month after preoperative (chemo)radiotherapy, but it is also the treatment that worries patients the most and contributes to increasing health anxiety and psychological distress after diagnosis. Psychological distress and coping style should therefore be monitored throughout the course of treatment, as at the end of active cancer treatments and at medium-term follow-up, psychological adjustment to the diagnosis of rectal cancer appears to explain HRQoL more than physical symptoms. Psychological programs should therefore promote the early adoption of active coping styles and prevent psychological distress to achieve better HRQoL in the medium term.

### CRediT authorship contribution statement

**Valentina Tesio:** Conceptualization, Formal analysis, Funding acquisition, Writing – original draft, Writing – review & editing. **Agata Benfante:** Data curation, Formal analysis, Project administration, Writing – original draft, Writing – review & editing. **Pierfrancesco Franco:** Conceptualization, Funding acquisition, Supervision, Writing – review & editing. **Annunziata Romeo:** Conceptualization, Methodology, Writing – review & editing. **Francesca Arcadipane:** Data curation, Methodology, Writing – review & editing. **Giuseppe Carlo Iorio:** Data curation, Project administration, Writing – review & editing. **Sara Bartoncini:** Data curation, Project administration, Writing – review & editing. **Lorys Castelli:** Conceptualization, Methodology, Supervision, Writing – review & editing.

## Funding

Valentina Tesio received a funding for the project by Fondazione Cassa di Risparmio di Torino (Project 2018.2324).

## Declaration of competing interest

The authors declare that they have no known competing financial interests or personal relationships that could have appeared to influence the work reported in this paper.

## References

[1] Sung H., Ferlay J., Siegel R.L. (2021). Global cancer statistics 2020: GLOBOCAN estimates of
incidence and mortality worldwide for 36 cancers in 185
countries. CA Cancer J Clin.

[2] Glynne-Jones R., Wyrwicz L., Tiret E., Brown G., Rödel C., Cervantes A., Arnold D., ESMO Guidelines Committee (2017). Rectal cancer: ESMO clinical practice guidelines for
diagnosis, treatment and follow-up. Ann Oncol.

[3] Mahmoud N.N. (2022). Colorectal cancer: preoperative evaluation and
staging. Surg Oncol Clin N Am.

[4] Haas S., Mikkelsen A.H., Kronborg C.J.S. (2023). Management of treatment-related sequelae following
colorectal cancer. Colorectal Dis.

[5] Simillis C., Khatri A., Dai N. (2023). A systematic review and network meta-analysis of
randomised controlled trials comparing neoadjuvant treatment
strategies for stage II and III rectal cancer. Crit Rev Oncol Hematol.

[6] Bours M.J., van der Linden B.W., Winkels R.M. (2016). Candidate predictors of health-related quality of life
of colorectal cancer survivors: a systematic
review. Oncologist.

[7] Fernández-Martínez D., Rodríguez-Infante A., Otero-Díez J.L., Baldonedo-Cernuda R.F., Mosteiro-Díaz M.P., García-Flórez L.J. (2020). Is my life going to change? A review of quality of
life after rectal resection. J Gastrointest Oncol.

[8] Murata A., Brown C.J., Raval M., Phang P.T. (2008). Impact of short-course radiotherapy and low anterior
resection on quality of life and bowel function in primary
rectal cancer. Am J Surg.

[9] Neibart S.S., Manne S.L., Jabbour S.K. (2020). Quality of life after radiotherapy for rectal and anal
cancer. Curr Colorectal Cancer Rep.

[10] Sales P.M., Carvalho A.F., McIntyre R.S., Pavlidis N., Hyphantis T.N. (2014). Psychosocial predictors of health outcomes in
colorectal cancer: a comprehensive review. Cancer Treat Rev.

[11] Deng M., Lan Y., Luo S. (2013). Quality of life estimate in stomach, colon, and rectal
cancer patients in a hospital in China. Tumour Biol.

[12] Acquati C., Hendren S., Wittmann D. (2022). Psychological and sexual distress in rectal cancer
patients and partners. Psychooncology.

[13] Rades D., Al-Salool A., Yu N.Y., Bartscht T. (2023). Emotional distress prior to chemoradiation for rectal
or anal cancer. In Vivo.

[14] De Vries A.M., Forni V., Voellinger R., Stiefel F. (2012). Alexithymia in cancer patients: review of the
literature. Psychother Psychosom.

[15] Voogt E., van der Heide A., van Leeuwen A.F. (2005). Positive and negative affect after diagnosis of
advanced cancer. Psychooncology.

[16] Di Tella M., Benfante A., Airale L., Castelli L., Milan A. (2023). Alexithymia and hypertension: does personality matter?
a systematic review and meta-analysis. Curr Cardiol Rep.

[17] Taylor G.J., Bagby R.M., Parker J.D. (1999).

[18] Lazarus R.S., Folkman S. (1987). Transactional theory and research on emotions and
coping. Eur J Pers.

[19] Lashbrook M.P., Valery P.C., Knott V., Kirshbaum M.N., Bernardes C.M. (2018). Coping strategies used by breast, prostate, and
colorectal cancer survivors: a literature review. Cancer Nurs.

[20] Kang Y., Son H. (2019). Age differences in the coping strategies of patients
with colorectal cancer. Cancer Nurs.

[21] Cicero V., Lo Coco G., Gullo S., Lo V.G. (2009). The role of attachment dimensions and perceived social
support in predicting adjustment to cancer. Psychooncology.

[22] Haviland J., Sodergren S., Calman L. (2017). Social support following diagnosis and treatment for
colorectal cancer and associations with health-related quality
of life: Results from the UK ColoREctal Wellbeing (CREW) cohort
study. Psychooncology.

[23] Fokas E., Schlenska-Lange A., Polat B. (2022). Chemoradiotherapy plus induction or consolidation
chemotherapy as total neoadjuvant therapy for patients with
locally advanced rectal cancer: long-term results of the
CAO/ARO/AIO-12 randomized clinical trial. JAMA Oncol.

[24] Kosmala R., Fokas E., Flentje M. (2021). Quality of life in rectal cancer patients with or
without oxaliplatin in the randomised CAO/ARO/AIO-04 phase 3
trial. Eur J Cancer.

[25] Aaronson N.K., Ahmedzai S., Bergman B. (1993). The European organization for research and treatment
of cancer QLQ-C30: a quality-of-life instrument for use in
international clinical trials in oncology. J Natl Cancer Inst.

[26] Whistance R.N., Conroy T., Chie W. (2009). Clinical and psychometric validation of the EORTC
QLQ-CR29 questionnaire module to assess health-related quality
of life in patients with colorectal cancer. Eur J Cancer.

[27] Bjelland I., Dahl A.A., Haug T.T., Neckelmann D. (2002). The validity of the Hospital Anxiety and Depression
Scale. An updated literature review. J Psychosom Res.

[28] Zigmond A.S., Snaith R.P. (1983). The hospital anxiety and depression
scale. Acta Psychiatr Scand.

[29] Watson D., Clark L.A., Tellegen A. (1988). Development and validation of brief measures of
positive and negative affect: the PANAS scales. J Pers Soc Psychol.

[30] Taylor G.J., Bagby R.M., Parker J.D. (2003). The 20-Item Toronto Alexithymia Scale. IV. Reliability
and factorial validity in different languages and
cultures. J Psychosom Res.

[31] Watson M., Law M., dos Santos M., Greer S., Baruch J., Bliss J. (1994). The Mini-MAC: further development of the mental
adjustment to cancer scale. J Psychosoc Oncol.

[32] Zimet G.D., Powell S.S., Farley G.K., Werkman S., Berkoff K.A. (1990). Psychometric characteristics of the multidimensional
scale of perceived social support. J Pers Assess.

[33] Orive M., Anton-Ladislao A., Lázaro S. (2022). Anxiety, depression, health-related quality of life,
and mortality among colorectal patients: 5-year
follow-up. Support Care Cancer.

[34] Qaderi S.M., van der Heijden J.A.G., Verhoeven R.H.A., de Wilt J.H.W., Custers J.A.E., PLCRC study group (2021). Trajectories of health-related quality of life and
psychological distress in patients with colorectal cancer: A
population-based study. Eur J Cancer.

[35] Stiegelis H.E., Ranchor A.V., Sanderman R. (2004). Psychological functioning in cancer patients treated
with radiotherapy. Patient Educ Couns.

[36] Hernández Blázquez M., Cruzado J.A. (2016). A longitudinal study on anxiety, depressive and
adjustment disorder, suicide ideation and symptoms of emotional
distress in patients with cancer undergoing
radiotherapy. J Psychosom Res.

[37] Wang I.Y., Jane S.W., Hsu H.C. (2023). The longitudinal trends of care needs, psychological
distress, and quality of life and related predictors in
Taiwanese colorectal cancer survivors. Semin Oncol Nurs.

[38] Reudink M., Molenaar C.J.L., Bonhof C.S., Janssen L., Mols F., Slooter G.D. (2022). Evaluating the longitudinal effect of colorectal
surgery on health-related quality of life in patients with
colorectal cancer. J Surg Oncol.

[39] Mosher C.E., Winger J.G., Given B.A., Shahda S., Helft P.R. (2017). A systematic review of psychosocial interventions for
colorectal cancer patients. Support Care Cancer.

[40] Grimmett C., Heneka N., Chambers S. (2022). Psychological interventions prior to cancer surgery: a
review of reviews. Curr Anesthesiol Rep.

