# Supplementary material for: The time course of health-related Quality of Life in rectal cancer patients undergoing combined modality treatment
Source: Clin Transl Radiat Oncol. 2024 Jul 26;48:100824. doi: 10.1016/j.ctro.2024.100824 (PMC11332791; doi:10.1016/j.ctro.2024.100824)
Supplement: Supplementary Data 1 [file mmc1.docx]

***Supplemental Appendix 1.*** *Pearson correlations among Health-Related Quality of life (QLQ-C30) at the different time points (T0, T1, T2 and T3 – follow-up) and all other variables.*

|  |  | **QLQ-C30_T0**  **N = 43** | **QLQ-C30_T1**  **N = 40** | | **QLQ-C30_T2**  **N = 37** | | | **QLQ-C30_T3**  **N = 31** | | | |
| --- | --- | --- | --- | --- | --- | --- | --- | --- | --- | --- | --- |
| **Variable assessed at time:** | | **T0** | **T0** | **T1** | **T0** | **T1** | **T2** | **T0** | **T1** | **T2** | **T3** |
| **Age** | | 0.118 | 0.027 | / | 0.105 | / | / | 0.109 | / | / | / |
| **Gender** | | –0.131 | –0.146 | / | –0.021 | / | / | –0.200 | / | / | / |
| **Years of education** | | 0.159 | 0.297 | / | –0.053 | / | / | –0.283 | / | / | / |
| **QLQ-CR29** | |  |  |  |  |  |  |  |  |  |  |
|  | QLQ-CR29_BI | 0.366^*^ | 0.529^***^ | 0.618^***^ | 0.230 | 0.256 | 0.289 | 0.151 | 0.238 | 0.313 | 0.536** |
|  | QLQ-CR29_Anx | 0.401^**^ | 0.263 | 0.381^*^ | 0.099 | 0.173 | 0.578^***^ | 0.175 | 0.389^*^ | 0.129 | 0.398* |
|  | QLQ-CR29_Wei | 0.197 | 0.309 | 0.267 | 0.019 | 0.023 | 0.312 | 0.085 | 0.146 | 0.378^*^ | 0.090 |
|  | QLQ-CR29_SexInt | 0.057 | 0.175 | 0.279 | 0.158 | 0.100 | 0.093 | 0.118 | 0.158 | 0.347 | 0.194 |
|  | QLQ-CR29_SexSy | –0.024 | 0.051 | –0.235 | –0.064 | –0.142 | –0.125 | –0.294 | –0.301 | –0.341 | –0.005 |
|  | QLQ-CR29_UrSy | –0.499^***^ | –0.600^***^ | –0.643^***^ | –0.251 | –0.434^**^ | –0.294 | –0.018 | –0.009 | –0.014 | –0.181 |
|  | QLQ-CR29_InSy | –0.734^***^ | –0.668^***^ | –0.777^***^ | –0.184 | –0.182 | –0.372^*^ | –0.289 | –0.229 | –0.339 | –0.318 |
|  | QLQ-CR29_PainSy | –0.633^***^ | –0.738^***^ | –0.575^***^ | –0.246 | –0.494^**^ | –0.420^**^ | –0.328 | –0.150 | –0.344 | –0.724^**^ |
|  | QLQ-CR29_MoSy | –0.544^***^ | –0.402^*^ | –0.497^***^ | –0.241 | –0.435^**^ | –0.664^***^ | –0.213 | –0.140 | –0.335 | –0.536^**^ |
| **TAS-20** | | –0.207 | –0.297 | / | –0.419^**^ | / | / | –0.125 | / | / | / |
| **PANAS** | |  |  |  |  |  |  |  |  |  |  |
|  | PANAS_PAtr | 0.102 | 0.014 | / | 0.387^*^ | / | / | 0.173 | / | / | / |
|  | PANAS_NAtr | –0.389^**^ | –0.252 | / | –0.189 | / | / | 0.173 | / | / | / |
|  | PANAS_PAst | 0.114 | 0.242 | 0.432^**^ | 0.258 | 0.311 | 0.541^***^ | 0.107 | 0.552^**^ | 0.486^**^ | 0.662^**^ |
|  | PANAS_NAst | –0.236 | –0.069 | –0.372^*^ | –0.374^*^ | –0.325 | –0.388^*^ | 0.203 | –0.078 | –0.152 | –0.271 |
| **HADS** | | –0.581^***^ | –0.402^*^ | –0.631^***^ | –0.306 | –0.384^*^ | –0.743^***^ | 0.017 | –0.242 | –0.299 | –0.615^**^ |
| **MSPSS** | | 0.098 | 0.233 | 0.140 | 0.208 | 0.162 | 0.220 | –0.012 | –0.092 | –0.009 | 0.069 |
| **Mini-MAC** | |  |  |  |  |  |  |  |  |  |  |
|  | Mini-MAC_F | <0.001 | –0.205 | –0.110 | 0.121 | 0.347^*^ | 0.310 | 0.028 | 0.400^*^ | 0.166 | 0.075 |
|  | Mini-MAC_FS | 0.029 | –0.222 | –0.024 | –0.042 | 0.316 | 0.386^*^ | –0.145 | 0.438^*^ | 0.209 | 0.203 |
|  | Mini-MAC_HH | –0.132 | –0.156 | –0.562^***^ | –0.036 | –0.101 | –0.487^**^ | 0.047 | –0.170 | –0.272 | –0.322 |
|  | Mini-MAC_AP | –0.239 | –0.223 | –0.631^***^ | –0.259 | –0.256 | –0.461^**^ | –0.063 | –0.226 | –0.337 | –0.414^*^ |
|  | Mini-MAC_CA | –0.023 | –0.270 | –0.401^*^ | –0.106 | 0.003 | –0.027 | –0.247 | –0.064 | –0.247 | –0.250 |

**p-value < 0.05; **p-value < 0.01; ***p-value < 0.001.*

*QLQ-C30: EORTC quality of life questionnaire; QLQ-CR29: EORTC colorectal cancer module: _BI: Body Image. _Anx: Anxiety. _Wei: Weight. _SexInt: Sexual Interest. functional scales; _SexSy: Sexual Symptoms. _UrSy: Urinary Symptoms. _InSy: Intestinal Symptoms. _PainSy: Pain Symptoms. _MoSy: Mouth Symptoms. subcales; TAS-20: Toronto Alexithymia Scale; PANAS: Positive and Negative Affect Scale. _PAtr: Positive Affect trait; _NAtr: Negative Affect trait; _PAst: Positive Affect state; _NAst: Negative Affect state; HADS: Hospital Anxiety and Depression Scale; MSPSS: Multidimensional Scale of Perceived Social Support; Mini-MAC: Mini-Mental Adjustment to Cancer Scales. _F: Fatalism. _FS: Fighting Spirit. _HH: Helplessness/Hopelessness. _AP: Anxious Preoccupation. _CA: Cognitive Avoidance.*

***Supplemental Appendix 2.*** *Hierarchical multiple* *regression with Health-Related Quality of Life (QLQ-C30) at T0 as dependent variable (N = 43).*

|  | **Predictor** | **R^2^** | **Adj R^2^** | **F** | **F- ΔR^2^** | **B** | **SE B** | ***β*** | ***p*** |
| --- | --- | --- | --- | --- | --- | --- | --- | --- | --- |
| **1** | *(Constant)* | 0.54 | 0.53 | 47.78*** | 47.78*** | *95.07* | *1.51* |  | *<0.001* |
|  | QLQ-CR29_InSy_T0 |  |  |  |  | –0.48 | 0.069 | –0.734 | <0.001 |
| **2** | *(Constant)* | 0.63 | 0.61 | 33.77*** | 9.66** | *96.65* | *1.462* |  | *<0.001* |
|  | QLQ-CR29_InSy_T0 |  |  |  |  | –0.36 | 0.073 | –0.555 | <0.001 |
|  | QLQ-CR29_PainSy_T0 |  |  |  |  | –0.18 | 0.059 | –0.349 | 0.003 |
| **3** | *(Constant)* | 0.67 | 0.65 | 26.65*** | 5.25* | *92.69* | *2.218* |  | *<0.001* |
|  | QLQ-CR29_InSy_T0 |  |  |  |  | –0.36 | 0.069 | –0.552 | <0.001 |
|  | QLQ-CR29_PainSy_T0 |  |  |  |  | –0.15 | 0.058 | –0.282 | 0.015 |
|  | QLQ-CR29_Anx_T0 |  |  |  |  | 0.07 | 0.030 | 0.221 | 0.027 |
| **4** | *(Constant)* | 0.71 | 0.68 | 23.33*** | 5.05* | *98.93* | *3.49* |  | *<0.001* |
|  | QLQ-CR29_InSy_T0 |  |  |  |  | –0.38 | 0.07 | –0.581 | <0.001 |
|  | QLQ-CR29_PainSy_T0 |  |  |  |  | –0.11 | 0.06 | –0.199 | 0.082 |
|  | QLQ-CR29_Anx_T0 |  |  |  |  | 0.06 | 0.03 | 0.185 | 0.054 |
|  | PANAS_NAtr_T0 |  |  |  |  | –0.33 | 0.15 | –0.215 | 0.030 |

**p-value < 0.05; **p-value < 0.01; ***p-value < 0.001*

*QLQ-C30: EORTC quality of life questionnaire; QLQ-CR29: EORTC colorectal cancer module: _InSy: Intestinal Symptoms. _PainSy: Pain Symptoms. _Anx: Anxiety. subscales; PANAS_NAtr: Positive and Negative Affect Scale_Negative Affect trait.*

***Supplemental Appendix 3.*** *Hierarchical multiple* *regression with Health-Related Quality of Life (QLQ-C30) at T1 as dependent variable (N = 40).*

|  | **Predictor** | **R^2^** | **Adj R^2^** | **F** | **F- ΔR^2^** | **B** | **SE B** | ***β*** | ***p*** |
| --- | --- | --- | --- | --- | --- | --- | --- | --- | --- |
| **1** | *(Constant)* | 0.55 | 0.53 | 45.49*** | 45.49*** | *97.57* | *2.03* |  | *<0.001* |
|  | QLQ-CR29_PainSy_T0 |  |  |  |  | –0.53 | 0.08 | –0.738 | <0.001 |
| **2** | *(Constant)* | 0.70 | 0.68 | 43.24*** | 19.20** | *99.47* | *1.73* |  | *<0.001* |
|  | QLQ-CR29_PainSy_T0 |  |  |  |  | –0.44 | 0.07 | –0.613 | <0.001 |
|  | QLQ-CR29_UrSy_T0 |  |  |  |  | –0.39 | 0.09 | –0.414 | <0.001 |
| **3** | *(Constant)* | 0.79 | 0.77 | 44*** | 14.33*** | *100.13* | *1.49* |  | *<0.001* |
|  | QLQ-CR29_PainSy_T0 |  |  |  |  | –0.31 | 0.07 | –0.436 | <0.001 |
|  | QLQ-CR29_UrSy_T0 |  |  |  |  | –0.25 | 0.09 | –0.262 | 0.006 |
|  | QLQ-CR29_InSy_T1 |  |  |  |  | –0.38 | 0.10 | –0.395 | <0.001 |
| **4** | *(Constant)* | 0.82 | 0.80 | 40.93*** | 7.58** | *101.17* | *1.42* |  | *<0.001* |
|  | QLQ-CR29_PainSy_T0 |  |  |  |  | –0.33 | 0.06 | –0.458 | <0.001 |
|  | QLQ-CR29_UrSy_T0 |  |  |  |  | –0.06 | 0.11 | –0.063 | 0.569 |
|  | QLQ-CR29_InSy_T1 |  |  |  |  | –0.31 | 0.10 | –0.322 | 0.003 |
|  | QLQ-CR29_UrSy_T1 |  |  |  |  | –0.25 | 0.09 | –0.306 | 0.009 |

***p-value < 0.01; ***p-value < 0.001*

*QLQ-C30: EORTC quality of life questionnaire; QLQ-CR29: EORTC colorectal cancer module: _PainSy: Pain Symptoms; _UrSy: Urinary Symptoms. _InSy: Intestinal Symptoms. subscales.*

***Supplemental Appendix 4.*** *Hierarchical multiple* *regression with Health-Related Quality of Life (QLQ-C30) at T2 as dependent variable (N = 37).*

|  | **Predictor** | **R^2^** | **Adj R^2^** | **F** | **F- ΔR^2^** | **B** | **SE B** | ***β*** | ***p*** |
| --- | --- | --- | --- | --- | --- | --- | --- | --- | --- |
| **1** | *(Constant)* | 0.18 | 0.15 | 7.44** | 7.44* | 103.41 | 8.61 |  | *<0.001* |
|  | TAS-20 |  |  |  |  | –0.52 | 0.19 | –0.419 | 0.010 |
| **2** | *(Constant)* | 0.34 | 0.30 | 8.56*** | 8.15** | 119.46 | 9.66 |  | *<0.001* |
|  | TAS-20 |  |  |  |  | –0.55 | 0.17 | –0.442 | 0.003 |
|  | PANAS_NAst_T0 |  |  |  |  | –0.81 | 0.28 | –0.400 | 0.007 |
| **3** | *(Constant)* | 0.45 | 0.40 | 9.10*** | 7.11* | 97.63 | 12.09 |  | *<0.001* |
|  | TAS-20 |  |  |  |  | –0.55 | 0.16 | –0.446 | 0.002 |
|  | PANAS_NAst_T0 |  |  |  |  | –0.79 | 0.26 | –0.393 | 0.005 |
|  | Mini-MAC_F_T1 |  |  |  |  | 7.36 | 2.76 | 0.343 | 0.012 |
| **4** | *(Constant)* | 0.67 | 0.63 | 16.17*** | 20.92*** | 95.84 | 9.55 |  | *<0.001* |
|  | TAS-20 |  |  |  |  | –0.36 | 0.13 | –0.286 | 0.012 |
|  | PANAS_NAst_T0 |  |  |  |  | –0.65 | 0.21 | –0.323 | 0.004 |
|  | Mini-MAC_F_T1 |  |  |  |  | 5.97 | 2.20 | 0.279 | 0.011 |
|  | QLQ-CR29_MoSy_T2 |  |  |  |  | –0.32 | 0.07 | –0.500 | <0.001 |
| **5** | *(Constant)* | 0.75 | 0.71 | 18.97*** | 10.65** | 92.21 | 8.45 |  | *<0.001* |
|  | TAS-20 |  |  |  |  | –0.18 | 0.13 | –0.143 | 0.181 |
|  | PANAS_NAst_T0 |  |  |  |  | –0.39 | 0.20 | –0.195 | 0.056 |
|  | Mini-MAC_F_T1 |  |  |  |  | 5.15 | 1.95 | 0.240 | 0.013 |
|  | QLQ-CR29_MoSy_T2 |  |  |  |  | –0.23 | 0.07 | –0.369 | 0.001 |
|  | HADS_T2 |  |  |  |  | –0.81 | 0.25 | –0.394 | 0.003 |

**p-value < 0.05; **p-value < 0.01; ***p-value < 0.001*

*QLQ-C30: EORTC quality of life questionnaire; TAS-20: Toronto Alexithymia Scale; PANAS_NAst: Positive and Negative Affect Scale_Negative Affect state; QLQ-CR29: EORTC colorectal cancer module: _MoSy: Mouth Symptoms. subscale; Mini-MAC_F: Mini-Mental Adjustment to Cancer Scales_Fatalism; HADS: Hospital Anxiety and Depressive Scale.*

***Supplemental Appendix 5.*** *Hierarchical multiple* *regression with Health-Related Quality of Life (QLQ-C30) at follow-up (T3) as dependent variable (N = 31).*

|  | **Predictor** | **R^2^** | **Adj R^2^** | **F** | **F- ΔR^2^** | **B** | **SE B** | ***β*** | ***p*** |
| --- | --- | --- | --- | --- | --- | --- | --- | --- | --- |
| **1** | *(Constant)* | 0.15 | 0.12 | 5.17* | 5.17* | 76.975 | 4.854 |  | *<0.001* |
|  | QLQ-CR29_Anx_T1 |  |  |  |  | 0.159 | 0.07 | 0.389 | 0.031 |
| **2** | *(Constant)* | 0.40 | 0.36 | 9.49*** | 11.86** | 52.321 | 8.27 |  | *<0.001* |
|  | QLQ-CR29_Anx_T1 |  |  |  |  | 0.13 | 0.06 | 0.318 | 0.040 |
|  | PANAS_PAst_T1 |  |  |  |  | 0.85 | 0.247 | 0.508 | 0.002 |
| **3** | *(Constant)* | 0.49 | 0.42 | 8.5*** | 4.3* | 40.004 | 9.823 |  | *<0.001* |
|  | QLQ-CR29_Anx_T1 |  |  |  |  | 0.101 | 0.059 | 0.246 | 0.098 |
|  | PANAS_PAst_T1 |  |  |  |  | 0.824 | 0.234 | 0.492 | 0.002 |
|  | Mini-MAC_F_T1 |  |  |  |  | 5.04 | 2.431 | 0.296 | 0.048 |
| **4** | *(Constant)* | 0.70 | 0.65 | 14.94*** | 18.11*** | 62.925 | 9.385 |  | *<0.001* |
|  | QLQ-CR29_Anx_T1 |  |  |  |  | 0.049 | 0.048 | 0.121 | 0.309 |
|  | PANAS_PAst_T1 |  |  |  |  | 0.462 | 0.202 | 0.276 | 0.030 |
|  | Mini-MAC_F_T1 |  |  |  |  | 4.807 | 1.903 | 0.282 | 0.018 |
|  | QLQ-CR29_PainSy_T3 |  |  |  |  | –0.464 | 0.109 | –0.532 | <0.001 |
| **5** | *(Constant)* | 0.77 | 0.72 | 16.76*** | 7.99** | 66.359 | 8.42 |  | *<0.001* |
|  | QLQ-CR29_Anx_T1 |  |  |  |  | 0.059 | 0.042 | 0.143 | 0.178 |
|  | PANAS_PAst_T1 |  |  |  |  | 0.493 | 0.179 | 0.294 | 0.011 |
|  | Mini-MAC_F_T1 |  |  |  |  | 3.32 | 1.77 | 0.195 | 0.072 |
|  | QLQ-CR29_PainSy_T3 |  |  |  |  | –0.378 | 0.101 | –0.433 | <0.001 |
|  | QLQ-CR29_MoSy_T3 |  |  |  |  | –0.205 | 0.073 | –0.299 | 0.009 |
| **6** | *(Constant)* | 0.84 | 0.80 | 20.78*** | 10.16** | *81.67* | *8.66* |  | *<0.001* |
|  | QLQ-CR29_Anx_T1 |  |  |  |  | –0.02 | 0.04 | –0.053 | 0.627 |
|  | PANAS_PAst_T1 |  |  |  |  | 0.19 | 0.18 | 0.116 | 0.291 |
|  | Mini-MAC_F_T1 |  |  |  |  | 4.82 | 1.59 | 0.283 | 0.006 |
|  | QLQ-CR29_PainSy_T3 |  |  |  |  | –0.31 | 0.09 | –0.352 | 0.002 |
|  | QLQ-CR29_MoSy_T3 |  |  |  |  | –0.22 | 0.06 | –0.322 | 0.002 |
|  | HADS_T3 |  |  |  |  | –0.74 | 0.23 | –0.414 | 0.004 |

**p-value < 0.05; **p-value < 0.01; ***p-value < 0.001*

*QLQ-C30: EORTC quality of life questionnaire; PANAS_PAst: Positive and Negative Affect Scale_Positive Affect state; Mini-MAC_F: Mini-Mental Adjustment to Cancer Scales_Fatalism; QLQ-CR29: EORTC colorectal cancer module: _PainSy: Pain Symptoms; _MoSy: Mouth Symptoms. subscale; HADS: Hospital Anxiety and Depressive Scale.*
